# Parent of origin influences the cardiac expression of vascular endothelial growth factor (*Vegfa*)

**DOI:** 10.1186/1471-2350-14-43

**Published:** 2013-04-05

**Authors:** Christine L Chiu, Chloe T Morgan, Samantha J Lupton, Joanne M Lind

**Affiliations:** 1 University of Western Sydney, School of Medicine, Penrith, NSW 2751, Locked Bag 1797, Australia

**Keywords:** Parent of origin, Epigenetics, Vascular endothelial growth factor

## Abstract

**Background:**

Vascular endothelial growth factor A (VEGFA) is a major regulator of both physiological and pathological angiogenesis. Associations between polymorphisms in *VEGFA* and complex disease have been inconsistent. The parent from whom the allele was inherited may account for these inconsistencies. This study examined the parent of origin effect on the expression of murine *Vegfa*.

**Methods:**

Two homozygous, inbred mouse strains A/J (AJ) and 129x1/SvJ (129) were crossed to produce reciprocal AJ129 and 129AJ offspring, respectively. RNA was extracted from cardiac tissue of 6 week old male (n = 8) and female (n = 8) parental, and male and female F1 offspring mice (AJ129 n = 8 and 129AJ n = 8). *Vegfa* and *Hif1a* expression levels were measured by qPCR and compared between the F1 offspring from the reciprocal crosses.

**Results:**

We found significant differences in the expression of *Vegfa* in F1 offspring (AJ129 and 129AJ mice) of the reciprocal crosses between AJ and 129 mice. Offspring of male AJ mice had significantly higher expression of *Vegfa* than offspring of male 129 mice (p = 0.006). This difference in expression was not the result of preferential allele expression (allelic imbalance). Expression of *Hif1a*, a transcriptional regulator of *Vegfa* expression, was also higher in F1 offspring of an AJ father (p = 0.004).

**Conclusion:**

Differences in *Vegfa* and *Hif1a* gene expression are likely the result of an upstream angiogenic regulator gene that is influenced by the parent of origin. These results highlight the importance of including inheritance information, such as parent of origin, when undertaking allelic association studies.

## Background

Angiogenesis is the growth of capillary sized vessels. It plays a critical role in wound healing and embryological development, and in the pathogenesis of diseases such as cancer. Promoting angiogenesis can aid in the treatment of ischemia and myocardial infarction, while inhibition of angiogenesis has been effective in the treatment of cancer. Vascular endothelial growth factor A (VEGFA) is one of the most potent inducers of angiogenesis, and is a major regulator of both physiological and pathological angiogenesis. It has been shown to stimulate blood vessel formation in animal models of ischemic cardiovascular disease [1], has been trialled as a potential therapeutic for myocardial ischemia [2], and therapies that inhibit VEGF are used in the treatment of cancer [3].

A number of studies have investigated associations between polymorphisms within, or nearby, the *VEGFA* gene and complex disease, including coronary artery disease [4], atherosclerosis [5], myocardial infarction [6], and hypertension [7]. However, these sequence variants explain only a small fraction of the estimated heritability of the diseases studied, and the ability to replicate these findings can prove difficult. For example, inconsistent results have been shown when studying the association between the development of coronary artery lesions in individuals with Kawasaki disease with *VEGFA* polymorphisms [8,9]. Population differences, study power, and choice of polymorphism may explain inconsistent results. Additionally, the parent from whom the variant was inherited may also contribute to the inability to replicate results across studies.

Parent of origin effects influence gene expression in offspring and result in the preferential expression of the paternally or maternally inherited allele. It is caused by a variety of mechanisms including imprinting, sex, epigenetic effects, and environmental influences during development [10]. In genetic studies, parent of origin effects are largely ignored; however, recent studies have found evidence of parent of origin effects in breast cancer [11], colorectal cancer [12], and type 2 diabetes [11]. These findings highlight the importance of investigating the effects of parent of origin on phenotype and disease susceptibility.

Due to the inconsistency in results when studying the association between *VEGFA* and disease, this study aimed to determine whether parent of origin effects influence the expression of *Vegfa*. This study was carried out in cardiac tissue from F1 hybrids resulting from reciprocal crosses of two inbred mouse strains.

## Methods

All experiments were carried out at the School of Medicine Animal Facility, University of Western Sydney and approved by the University of Western Sydney Animal Ethics Committee. Two homozygous, inbred mouse strains A/J (AJ) and 129x1/SvJ (129) were used. Crosses between AJ male and 129 female, and 129 male and AJ female produced reciprocal AJ129 and 129AJ offspring, respectively. Male (n = 8) and female (n = 8) mice from each of the parental strains were euthanised at six weeks of age by cervical dislocation. Male and female offspring were collected from each of the crosses (AJ129 n = 8 per sex and 129AJ n = 8 per sex). The hearts were excised, rinsed with PBS and flash frozen in LN_2_.

## Results

Heterozygous F1 hybrids (AJ129 and 129AJ) resulting from reciprocal crosses of two inbred homozygous strains (AJ and 129) were used in this study. Direct sequencing of the full *Vegfa* mRNA transcript in homozygous parental strains and heterozygous F1 offspring confirmed a single nucleotide polymorphism (SNP) at the 3^′^UTR of the gene (AJ = GG, 129 = AA, AJ129 and 129AJ = GA).

### *Vegfa* expression

**Figure 1:**
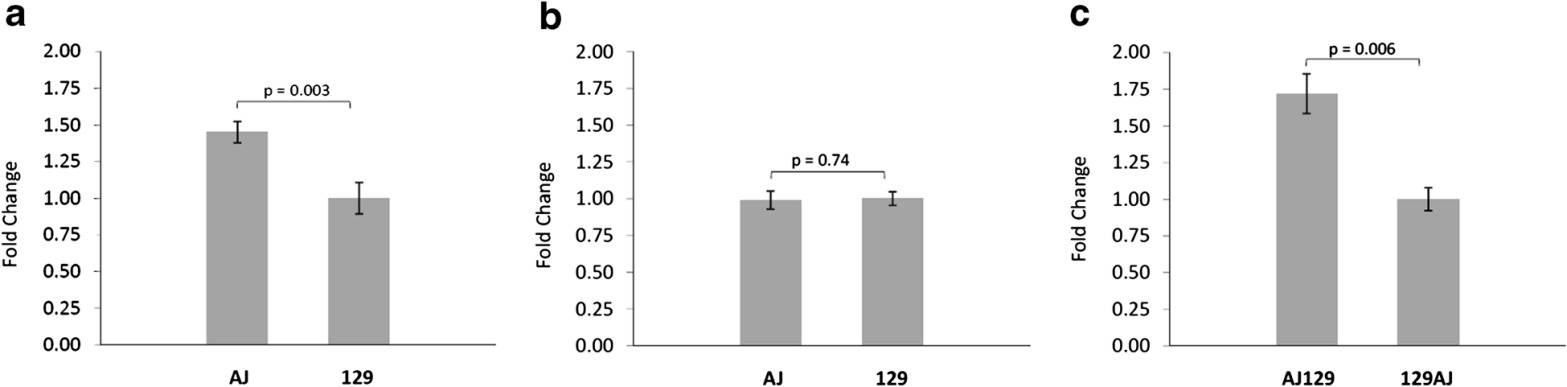
**Relative expression of *Vegfa.* a.** Parental: AJ male compared to 129 male. **b**. Parental: AJ female compared to 129 female. **c**. F1: AJ129 (offspring of AJ male) compared to 129AJ (offspring of 129 male).

This study compared the expression of *Vegfa* in AJ and 129 parental mice, and in male F1 offspring from reciprocal crosses (AJ129 and 129AJ), to determine whether parent of origin influences mRNA expression. Strain specific differences in *Vegfa* expression were seen between AJ and 129 mice, with *Vegfa* expression 1.5 fold higher in male AJ mice compared to male 129 mice (Figure 1a). No strain specific differences in *Vegfa* expression were observed between female AJ and female 129 mice (Figure 1b). In the male F1 offspring from the reciprocal crosses, *Vegfa* expression was 1.7 fold higher in AJ129 mice compared with 129AJ mice (Figure 1c). To ascertain whether this difference in *Vegfa* expression tracked with Y chromosome transmission, the expression of *Vegfa* was measured in female parentals and offspring. There was no difference in *Vegfa* expression between parental AJ males versus females (p = 0.15), and female offspring of AJ males had 1.3 fold increased expression of *Vegfa* (p = 0.03) compared with females of the reciprocal cross.

### Measurement of allelic expression

**Figure 2:**
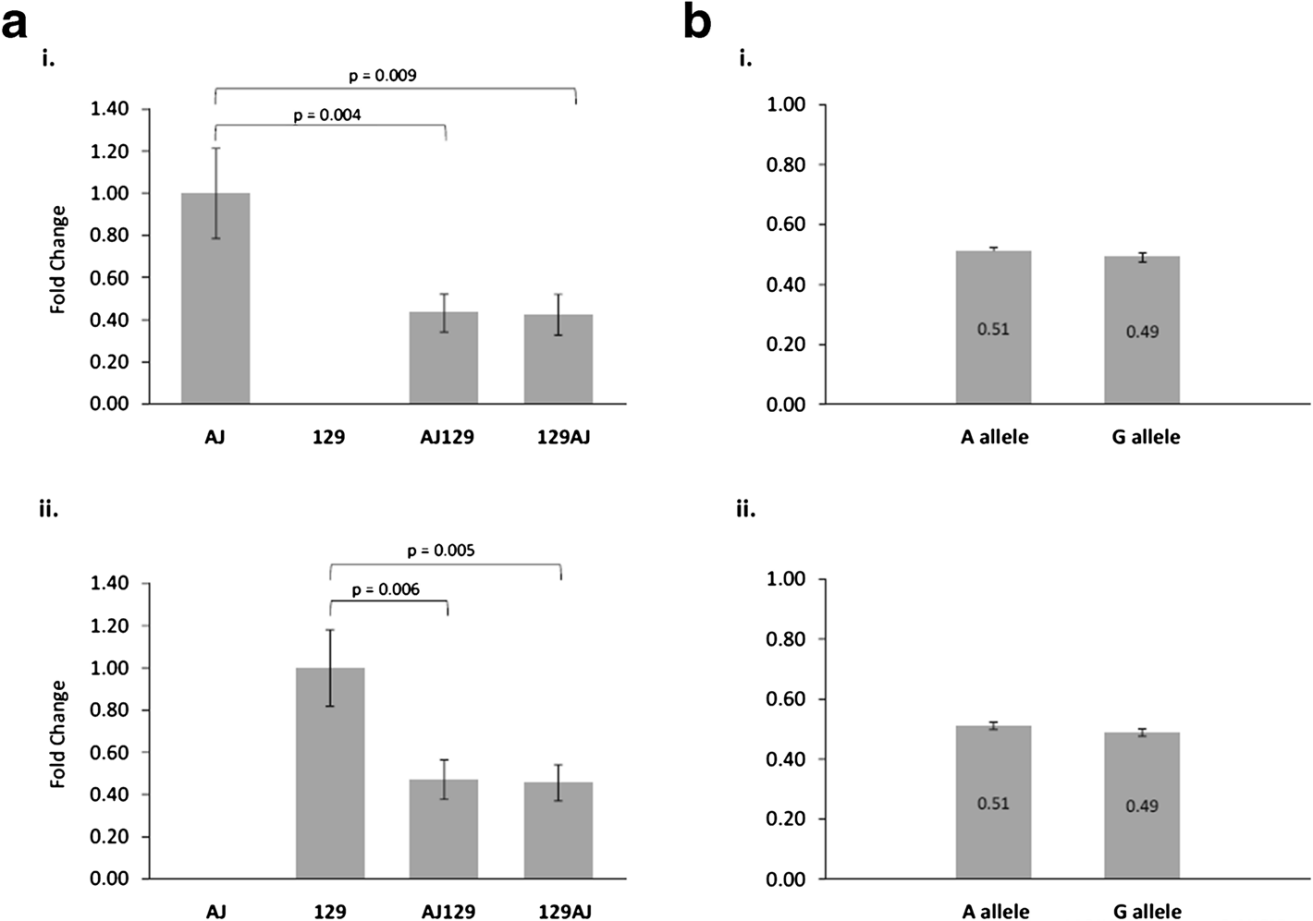
***Vegfa *allelic expression. ai.** Relative expression of the G allele in males. **aii**. Relative expression of the A allele in males. **bi**. Expression ratio of the G allele to the A allele in AJ129 mice. **bii**. Expression ratio of the G allele to the A allele in 129AJ mice.

To determine whether differences in *Vegfa* mRNA expression between the F1 offspring from the reciprocal crosses were the result of unequal expression of the maternally or paternally inherited allele, the expression of each allele was measured using quantitative ARMS PCR. The rs16821436 SNP in the 3^′^UTR of *Vegfa* (G/A) was used to differentiate expression of alleles in the F1 heterozygous mice. In the F1 offspring, the expression of each allele relative to the homozygous parental strain was significantly lower, as expected. The expression of the G allele in the heterozygous AJ129 and 129AJ mice was significantly lower, 43% and 43%, than the homozygous AJ mice (Figure 2ai). Similarly, the expression of the A allele in the heterozygous offspring was significantly lower, 47% and 45%, than the homozygous 129 parental mice (Figure 2aii). No difference in the expression of the G allele relative to the A allele was seen within the F1 offspring (Figure 2bi and ii). Additionally, there was no difference in the expression ratio of the G allele to the A allele between the AJ129 and 129AJ offspring.

### *Hif1a* expression

**Figure 3:**
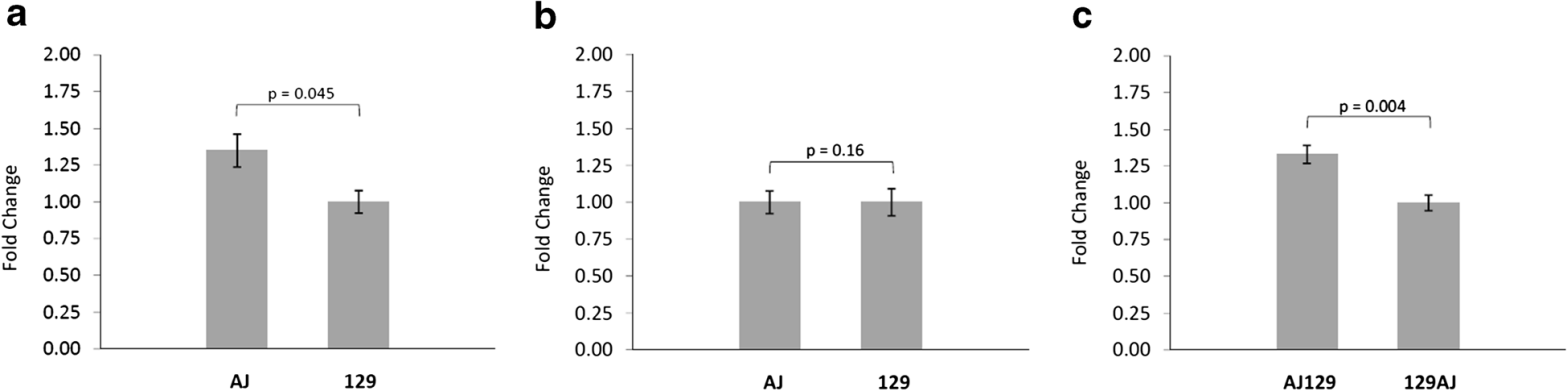
**Relative expression of *Hif1a.* a.** Parental: AJ male compared to 129 male. **b**. Parental: AJ female compared to 129 female. **c**. F1: AJ129 (offspring of AJ male) compared to 129AJ (offspring of 129 male).

To determine whether differences in *Vegfa* expression were the result of trans-acting regulatory elements, the expression of *Hif1a*, a transcription factor and known activator of *Vegfa* was measured. The expression of *Hif1a* was measured in the AJ and 129 parental strains and in the F1 offspring of reciprocal crosses. The expression of *Hif1a* was 1.4 fold higher in male AJ compared to male 129 mice (Figure 3a). No difference in *Hif1a* expression was observed between the female parental strains (Figure 3b). In the male F1 mice, the expression of *Hif1a* was 1.4 fold higher in AJ129 compared to 129AJ mice (Figure 3c).

## Discussion

This study examined the parent of origin effect on the expression of *Vegfa*, an important regulator of angiogenesis. We found significant differences in the expression of *Vegfa* in F1 offspring (AJ129 and 129AJ mice) of the reciprocal crosses between AJ and 129 mice. Specifically, offspring of male AJ mice had significantly higher expression of *Vegfa* than offspring of male 129 mice. This difference in expression was not the result of preferential allele expression (allelic imbalance), as no difference in the expression of the paternally or maternally inherited allele was observed in the F1 heterozygotes. Expression of *Hif1a*, a transcriptional regulator of *Vegfa* expression, was also found to be significantly higher in F1 offspring of an AJ father. These results cannot be explained by Y chromosome transmission, as female offspring of AJ fathers had increased expression of *Vegfa*. In addition, the AJ female parentals had no difference in expression of *Vegfa* when compared with the male AJ parentals. The DNA sequences of F1 heterozygotes are identical on autosomal chromosomes and differ only on their sex chromosomes. As *Vegfa* and *Hif1a* are not located on sex chromosomes, this suggests that the observed differences in *Vegfa* and *Hif1a* gene expression are not a result of DNA sequence variation and may be explained by a parent of origin effect.

Angiogenesis is a strictly regulated process that results from a balance between pro and inhibitory angiogenic factors. VEGF is a potent pro-angiogenic factor and its expression is tightly controlled at a number of levels. *Cis*-acting SNPs have been shown to alter *Vegfa* promoter activity [13-15], while SNPs in the 3^′^UTR have been reported to affect expression via the binding of micro RNAs which affect mRNA stability [16,17]. The AJ and 129 parental strains differed at a single nucleotide in the 3^′^UTR of *Vegf*[18]. In this study, the paternally and maternally inherited alleles within the F1 offspring were expressed equally, indicating that *cis*-acting regulatory elements within, or nearby, the *Vegfa* gene were unlikely to account for the observed differences in *Vegfa* gene expression. However, the presence of *cis*- acting regulatory elements in genes known to regulate *Vegfa* expression, which are influenced by parent of origin, may explain the difference in *Vegfa* expression.

A number of *trans*-acting factors are known to regulate the expression of *Vegfa*. Transcription factors that bind to response elements within the *Vegfa* promoter include hypoxia-inducible factor (HIF1) [19], nuclear factor (NF)-κB [20], activator protein (AP1) [21], and specificity protein (SP1) [22]. HIF1 is a transcription factor that functions to restore oxygen homeostasis during hypoxia via the regulation of genes involved in angiogenesis (*Vegfa*), glycolysis and vasodilation [23]. In this study, *Hif1a* expression was significantly higher in the F1 offspring of AJ males which corresponded to increased *Vegfa* expression within these same animals. Imbalanced expression of alleles within the *Hif1a* gene could not be assessed as the F1 mice were homozygous [18]. No studies have reported a role for epigenetic mechanisms in the regulation of *Hif1* expression however, epigenetic regulation of von Hippel-Lindau (VHL), an ubiquitin ligase that targets HIF1a for proteosomal destruction under normoxic conditions, has been reported. Hypermethylation of the *VHL* promoter in renal cell carcinoma has been correlated with increased *HIF1a* expression [24], and deletion of miR-23b has been reported to reduce *HIF1a* and *VEGFA* expression via its targeting of *VHL*[25]. Further work is needed to address whether epigenetic mechanisms are involved in the regulation of genes known to regulate *Vegfa* and *Hif1a* expression in this mouse model.

Transgenerational epigenetic inheritance is another mechanism that may explain the differences in expression patterns between the F1 offspring from the reciprocal crosses. It refers to genetic or environmental exposures in the parent, which affect patterns of gene expression in the offspring, without the offspring having inherited the allele or been exposed to the environmental agent [26]. Examples of transgenerational epigenetic inheritance include DNA methylation, histone modifications, changes in chromatin structure and the action of small non-coding RNA all of which can result in epialleles. That is, alleles that are variably expressed in the absence of genetic heterogeneity [27]. The epigenetic state is established during early embryogenesis and can be influenced by whether the allele is inherited from the mother or the father, as is the case with the agouti viable yellow allele (A^vy^) in mice [28]. Parent of origin effects may be observed for a number of genes, if these genes belong to pathways that are ultimately regulated by epialleles. This may be the case in the present study, where a regulator of genes involved in angiogenesis is influenced by the parent of origin, and downstream this alters the expression of both *Hif1a* and *Vegfa*. Further work is required to establish which genes are controlled by epigenetic mechanisms within the pathway controlling angiogenesis.

## Conclusions

Our study demonstrates that parent of origin influences *Vegfa* expression in mouse cardiac tissue, and the genetic factors which determine gene expression are more complex than simple Mendelian inheritance. Additionally, given the common use of A/J and 129x1/SvJ strains to generate genetically engineered mice, studies using mixed genetic background mice, or studies using mice after backcrossing, need to consider parent of origin in addition to genetic variation. Finally, the identification of epigenetic regulation in genes involved in angiogenesis has important implications in how future genome-wide association studies need to incorporate technologies, which detect epigenetic and parent of origin information, to provide more informative associations.

### RNA extraction, quantification and cDNA synthesis

Hearts were mechanically disrupted in the deep frozen state using the ‘Mikro Dismembrator’ automated frozen tissue disruptor (Sartorius, Germany), as previously described [29]. Total RNA was extracted from powderised tissue using TRIzol (Sigma Aldrich, USA) as per manufacturer’s protocol, followed by DNase I treatment using the ‘DNA-free’ kit (Applied Biosciences, USA) according to the manufacturer’s protocol. RNA were quantified using the NanoPhotometer (Implen, Germany) and cDNA was synthesised from 500 ng of RNA using Bioscript™ Reverse Transcriptase and random hexamers (Bioline Pty Ltd, Australia) according to the manufacturer’s protocol. Genomic DNA was extracted using the QIAamp DNA Mini Kit (Qiagen, Germany) as per the manufacturer’s protocol.

### Quantitative PCR

Quantitative PCR (qPCR) was used to measure mRNA expression of *Vegfa* and hypoxic inducible factor 1 alpha (*Hif1a*). Mitochondrial ribosomal protein S28 (*Mrps28S*), 18S ribosomal RNA (*Rn18S*), and hypoxanthine guanine phosphoribosyl transferase (*Hprt1*) were used as normalising genes. Primer sequences are described in Table 1. Individual reactions (10 μl) contained 5× MyTaq PCR buffer (Bioline), 0.4 U of MyTaq (Bioline), 1x SYBR Green I dye, forward and reverse primers (10 μM), and 4 μl of cDNA (or 4 μl water for no template controls). A pooled sample of cDNA was serially diluted 1 in 5 to generate a standard curve for each primer set, and the efficiency of the reaction was calculated. The PCR reactions were carried out in a MxPro3005P Real Time PCR System (Stratagene, Agilent Technologies, USA) under the conditions listed in Table 1, followed by a dissociation curve. All samples were run in triplicate.

**Table 1:** Primers and PCR conditions

**Primer**	**bp**	**Primer sequence 5**^**′ **^**to 3**^**′**^	**PCR conditions**
*Hprt1F*	94	TGACACTGGCAAAACAATGCA	95°C 10^′^ (95°C 40″; 60°C 30″; 72°C 30″) × 40
*Hprt1R*		GGTCCTTTTCACCAGCAAGCT	Dissociation curve
*Rn18SF*	178	CTCTGGTTGCTCTGTGCAGT	95°C 10^′^ (95°C 40″; 62°C 30″; 72°C 30″) × 40
*Rn18SR*		GGCTCCTTGTAGGGGTTCTC	Dissociation curve
*Mrps28SF*	223	GAAATGCAAGCACGGAGAGT	95°C 10^′^ (95°C 40″; 62°C 30″; 72°C 30″) × 40
*Mrps28SR*		CCGATGACCAGTTTGTCCTT	Dissociation curve
*VegfaF*	200	GCTACTGCCGTCCGATTGAGAC	95°C 10^′^ (95°C 40″; 65°C 30″; 72°C 30″) × 40
*VegfaR*		GTGCTGGCTTTGGTGAGGTTTG	Dissociation curve
*Hif1aF*	209	GCTTCTGTTATGAGGCTCACC	95°C 10^′^ (95°C 40″; 62°C 30″; 72°C 30″) × 40
*Hif1aR*		TCAAACTGAGTTAACCCCATGT	Dissociation curve

### Direct sequencing

Sanger sequencing was used to confirm the presence of a polymorphism in the 3^′^UTR of *Vegfa* (NCBI Reference Sequence: NM_001025250.3). Total RNA from the parental strains and F1 offspring was amplified and the purified PCR products were sequenced (Australian Genome Research Facility, AUST). Sequence data was aligned and analysed using Sequencher v5.0.1 (Gene Codes Corp, MI).

### Measurement of allelic expression using amplification refractory mutation systems (ARMS) PCR

Heterozygous F1 samples were evaluated for *Vegfa* allelic expression imbalance using quantitative ARMS PCR. The amplification primers (forward: CTTTCATCCCATTGTCTACC(G/A); reverse: TGTTATTGGTGTCTTCACTGGA) used in the assay generated a 188 bp amplicon. The allele specific primers were run in parallel reactions. Reactions were run under the following conditions: 95°C 10 min; 95°C 30 sec, 62°C 30 sec, 72°C 30 sec for 30 cycles; followed by a dissociation curve. Individual reactions (10 μl) contained 5× MyTaq PCR buffer (Bioline), 0.4 U of MyTaq (Bioline), 1x SYBR Green I dye, forward and reverse primers (10 μM), and 4 μl of cDNA (or 4 μl water for no template controls). All reactions were performed in triplicate. The ability of the primers to distinguish between the two alleles with high specificity was assessed using cDNA from parental strains. Under these conditions neither primer exhibited cross-reactivity with the opposite allele. A standard curve was generated for each primer pair using a dilution series (200 ng to 0.20 ng, 1 in 4) of heterozygous genomic DNA. This served as a reference for the 50:50 allelic ratio as we would expect a perfect 50:50 ratio of the two alleles in genomic DNA from a heterozygote with a diploid genome. We avoided using cDNA as a control as this would bias our results towards the allele expression ratio of the reference sample.

### Data analysis

For qPCR, cycle threshold (Ct) values for each sample were calculated using the MxPRo QPCR software (Stratagene, Agilent Technologies, USA). Triplicate Ct values were averaged and the quantity (Q) of each sample was calculated using the delta-delta Ct method [30]. Q values from the normaliser genes were input into geNorm [31] and the geometric means from these genes were used to generate a normalisation factor (NF). The Q values of the genes of interest were normalised by dividing by the NF value. Results are expressed as mean ± SE fold changes in expression.

Statistical significance between groups was determined using a general linear model. Statistical analysis was performed with PASW Statistics version 20. A *P* value of <0.01 was considered significant to account for multiple testing.

## Competing interests

The authors declare that they have no completing interest.

## Authors’ contributions

CLC carried out sample collection and preparation, gene expression studies, allele specific expression studies, data analysis and interpretation, and drafted the manuscript. CTM carried out sample collection and preparation, and gene expression studies. SJL carried out sample collection and preparation, and gene expression studies. JML conceived the study, participated in data analysis and interpretation, and helped to draft the manuscript. All authors read and approved the final manuscript.

## Pre-publication history

The pre-publication history for this paper can be accessed here:

http://www.biomedcentral.com/1471-2350/14/43/prepub
